# Evaluation of a multiplex PCR method to serotype *Salmonella* in animal feeds pre-enrichment broth cultures

**DOI:** 10.1016/j.mex.2017.09.003

**Published:** 2017-09-20

**Authors:** Junia Jean-Gilles Beaubrun, Laura Ewing, Kim Dudley, Faiza Benhamed, Hua Wang, Darcy E. Hanes

**Affiliations:** a U.S. Food and Drug Administration, Center for Food Safety and Applied Nutrition, Laurel, MD 207081, United States; b U.S. Food and Drug Administration, Center for Veterinary Medicine, Laurel, MD 20708, United States; c U.S. Food and Drug Administration, Center for Food Safety and Applied Nutrition, College Park, MD 20740, United States

**Keywords:** A molecular serotyping approach for *Salmonella* enterica detection in animal feed, *Salmonella* spp, molecular serotyping, animal feed

## Abstract

The identification of *Salmonella enterica* serotypes remains a highly important public health concern for microbiological analysis of foods, feeds, and clinical samples. Outbreaks of human salmonellosis are sometimes linked to contact with infected animals and animal feeds. To possibly reduce the number of outbreaks, it is important to rapidly, efficiently detect *Salmonella enterica* in animal feeds and food products. A multiplex PCR for molecular serotyping of *Salmonella enterica* previously used in a single lab validation study for serotyping in multiple human food matrices was used in this investigation to evaluate the effectiveness of the multiplex PCR assay as serotyping method and screening tool for *Salmonella* in animal feeds. This approach is unique in that:

• The multiplex PCR serotyping assay may be used for rapid screening and serotyping of *Salmonella enterica* from contaminated animal feed at the non-selective pre-enrichment step.
• The assay may provide the serotype or identification of *Salmonella* in positive samples at concentration as low as 10 CFU/25 g after a 24 h non-selective pre-enrichment step.
• In addition to the ability to serotype, this assay contains *inv*A as an internal control for *Salmonella* positive identification. The *inv*A shows positive indication for *Salmonella* outside of the 30 serotypic banding patterns.

## Graphical abstract

Screening, detection and serotyping of *Salmonella enterica* from 24 h pre-enrichment of animal feed.

**Figure:**
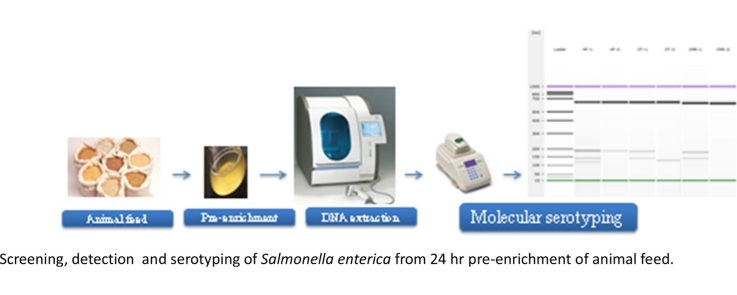

## Methods

### *Salmonella* identification in animal feed

Using a method described by Benhamed et al. [5] six feeds: Wheat Brand (WB), Horse Feed (HF), Dried Molasses (DM), Calf Milk Replacer (CMR), Dried Beet Pulp (DBP), and Whole Oats (OT) obtained from commercial sources were spiked with *S. enterica* serovar Typhimurium at concentrations of 10 CFU, 50 CFU, 100 CFU per 25 g, to evaluate the detection level using a modified version of the Bacteriological Analytical Manual (BAM), Chapter 5 [1]. Each sample was pre-enriched in Lactose broth (LB) and modified buffer peptone water (mBPW) media (n = 6 replicates for each medium). Two different media were compared to determine which media was more effective and if a specific media would be more efficient for the molecular assay [11].

**Fig. 1:**
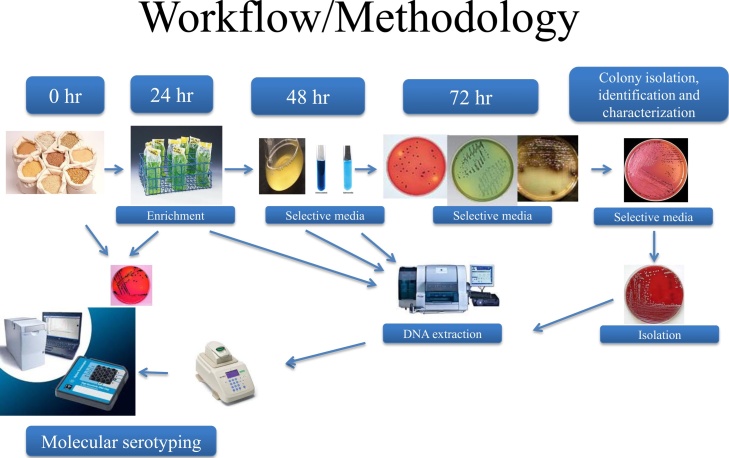
A graphical demonstration of the methodology for *Salmonella enterica* detection in animal feed.

Then HF, WB and CMR were spiked with 10 CFU/25 g and 2.5 CFU/25 g of *Salmonella enterica* serovars Typhimurium, Agona, and Hadar, respectively to evaluate the sensitivity of the molecular assay below the microbiological assay detection level of 10CFU/25 g. Each sample was pre-enriched in Lactose broth (LB) and modified buffer peptone water (mBPW) media (n = 20 replicates for each medium). The samples were prepared as described above and then aged for 2 weeks at 4 °C. A total of 92 samples were analyzed per feed type, 40 feed samples pre-enriched in lactose broth and 40 feed samples pre-enriched in mBPW. The 12 remaining samples were the positive controls of each serovar grown in each enrichment broth and un-inoculated enrichment broths were used as negative controls. The 24 h pre-enrichment broth cultures were then transferred into selective enrichment broths and selective plating followed by serological and biochemical confirmation, using a modified version of the BAM. Subsequently, recovered colonies were identified as *Salmonella* with Vitek^®^ 2 Compact, Version 5, (Biomerieux, St Louis, MO) [10]. Fig. 1 is a graphical demonstration of the methodology and workflow for *Salmonella* detection in animal feed.

### Major equipment and supplies for PCR assay

• Vitek^®^ 2 Compact, Version 5, (Biomerieux, St Louis, MO)
• Roche MagNA Pure Compact (Roche, Indianapolis IN)
• BioRad conventional C1000 thermocycler (BioRad, Hercules, CA)
• Eppendorf Centrifuge 5424R (Eppendorf, Hauppauge, NY)
• Agilent 2100 Bioanalyzer (Agilent Technologies, Waldbronn, Germany)
• DNA 1000 Reagents kit for DNA analysis (Agilent Technologies, Waldbronn, Germany)
• PCR tubes (BioRad, Hercules, CA))
• Sterile Eppendorf style microcentrifuge tubes ((Life Sciences, Hercules, CA or equivalent)
• Sterile inoculating loops or needles (Life Sciences, Hercules, CA or equivalent)
• Ice bucket or bench top cooler
• Adjustable Micropipettors (0.1–1000 μl)
• Aerosol resistant micropipettor tips (0.1–1000 μl)
• Vortex Mixer (Life Sciences, or equivalent)
• Microcentrifuge tips (Life Sciences, Hercules, CA or equivalent)

### Reagents for PCR assay

• Qiagen Hot Star Master Mix (Qiagen, Inc., Valencia, CA, 1000 U)
• PCR grade water (Life Sciences, Hercules, CA or equivalent)
• mM dNTP mix (Invitrogen Corporation, Carlsbad, CA, or equivalent)
• 10X Tris-Borate-EDTA Buffer (TBE) (Invitrogen Corporation, Carlsbad, CA)
• Primers (Integrated Technologies, IDT, San Diego, CA)
• mM working dilution of each primer mix from Table 2

### Molecular serotyping of *Salmonella*

#### DNA preparation

• One ml of samples after 24 h pre-enrichment. The samples were collected in a 1.5 ml sterile Eppendorf microcentrifudge tube.
• The tubes were centrifuged at 10,000 g for 10 min
• DNA was purified from the 24 h pellets using Roche MagNA Pure Compact (Roche, Indianapolis IN) according to the manufacturer’s instructions.
• DNA extraction quality and the PCR serotyping outcome are amplified by collecting samples at 24 h after inoculation. (Note: An important factor in the success of this assay is the ability to extract the DNA from the different matrices).
• The broth used for the pre-enrichment (Lactose or mBPW) showed no significant difference in recovery of *Salmonella*. Although the mBPW seems to be a better growth media for *Salmonella* in various matrices [11], in the animal feed it did not make a major difference.

##### PCR analysis

The PCR method consisted of two five-plex PCR reactions and one two-plex PCR reaction; the primers for each of the reactions are listed in Table 1, Table 2.

• The PCR master mix contained 5.0 μl of Q buffer, 5.0 μl of 10× Tris-Borate-EDTA buffer, 5.0 μl of 25 mM MgCl_2_ (Qiagen, Inc., Valencia, CA), 5.0 μl of a 10 mM concentration of the deoxynucleoside triphosphates (Invitrogen Corporation, Carlsbad, CA), 0.5 μl of HotStar Taq (Qiagen, Inc.) and 5.0 mM of each primer.
• PCR amplification of each sample was conducted using 2.0 μl of DNA template, and 48 μl of a master mix containing primers, for a total volume of 50 μl.
• The amplification parameters were as follows: a 5 min step at 94 °C, followed by 40 amplification cycles that consisted of a 30 s denaturing step at 94 °C, a 30 s annealing step at 56 °C, and a 1 min elongation step at 72 °C, and a final extension step of 5 min [11].
• Inhibitory factors may interfere with PCR results.
• Lack of *Salmonella* growth in this particular feed may also minimize detection by PCR
• Table 3 show that each feed type is different and the growth obtained from each feed also differed.

**Table 3:** A summary of the limit of detection study of the Horse Feed (HF), Whole Oats (OT), Dried Beet Pulp (DBP), Calf Milk Replacer (CMR), Dried Molasses (DM) and Wheat Brand (WB) spiked with 10 CFU/25 g, 50 CFU/25 g and 100 CFU/25 g of *Salmonella enterica* serovar Typhimurium.

Concentrations	HF	OT	DBP	CMR	DM	WB
100 cfu	+	+	−	+	+	+
50 cfu	+	+	−	+	+	+
10 cfu	−	+	−	±	−	+
Negative control	−	−	−	−	−	−

±represent positive *inv*A and negative serotypic patterns.

+represent positive serotypic pattern.

−represent negative serotypic pattern.

**Table 1:** Primers used in the PCR-Based Methods for serotyping *Salmonella enterica*.

Codes for the interpretation of the results.

**Table 2:** Preparation of Master Mix for Each Primer Set.

Component	Initial Concentration	Volume per 50 ul Reaction	Final Concentration
MASTERMIX for PRIMER SET 1			
Q Buffer		5 μl	
10X TBE Buffer	10X	5 μl	1 X
MgCl_2_	25 mM	5 μl	2.5 mM
dNTPs	10 mM	7 μl	1.4 mM
Primer Mix 1	5 mM	2.5 μl	0.25 mM
Hotstart	1000 U	0.5 μl	
Water		23.0 μl	
DNA template		2 μl	

MASTERMIX for PRIMER SET 2			
Q Buffer		5 μl	
10X TBE Buffer	10X	5 μl	1 X
MgCl_2_	25 mM	5 μl	2.5 mM
dNTPs	10 mM	7 μl	1.4 mM
Primer Mix 2	5 mM	2.5 μl	0.25 mM
Hotstart	1000 U	0.5 μl	
Water		23 μl	
DNA template		2 μl	

MASTERMIX for PRIMER SET 3			
Q Buffer		5 μl	
10X TBE Buffer	10X	5 μl	1 X
MgCl_2_	25 mM	5 μl	2.5 mM
dNTPs	10 mM	7 μl	1.4 mM
Primer Mix 3	5 mM	1 μl	.10 mM
Hotstart	1000 U	0.5 μl	
Water		24	
DNA template		2 μl	

##### Agilent data analysis

• PCR products were analyzed using the Agilent 2100 Bioanalyzer (Agilent Technologies, Waldbronn, Germany), and the DNA 1000 Reagents kit (Agilent Technologies) following the manufacturer’s protocol.
• Results were visualized as a gel image depicting the amplification of the PCR products (serotype-specific banding patterns) and the corresponding molecular weight for each PCR product.
• Serotypic banding patterns are represented by absence or presence of specific genes labeled A-L as shown in Table 1 [10].

**Fig. 2:**
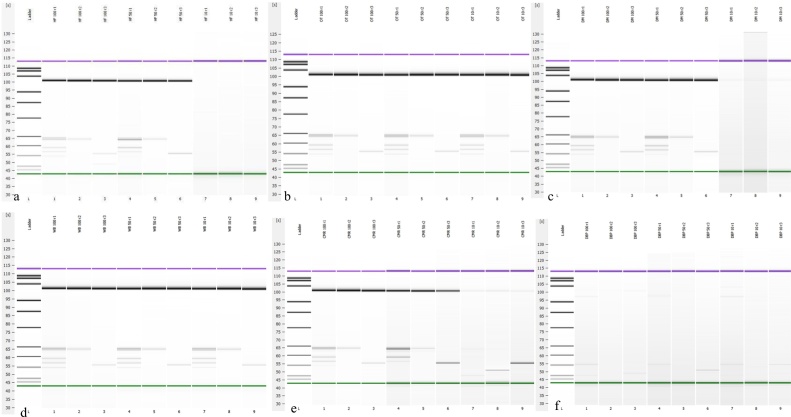
A representation of the gel image generated by the Agilent 2100 Bioanalyzer for multiplex PCR Serotyping method of *Salmonella enterica* serovar Typhimurium in Horse Feed (HF), Whole Oats (OT), Dried Beet Pulp (DBP), Calf Milk Replacer (CMR), Dried Molasses (DM) and Wheat Brand (WB) spiked with 10 CFU/25 g, 50 CFU/25 g and 100 CFU/25 g. Fig. 1a–f (Lane 1) Ladder, (Lane 2–4) 100 CFU/25 g of reaction 1–3, (Lane 5–7) 50 CFU/25 g of reaction 1–3, (Lane 8–10) 10 CFU/25 g of reaction 1–3. Figure a is for HF, Figure b is for OT, Figure c is for DM, Figure d is for WB, Figure e is for CMR, and Figure f is for DBP.

The multiplex PCR serotyping assay was performed concurrently with the *Bacteriological Analytical Method* (BAM). The optimal limit of detection in all the feeds was 50 CFU/25 g and lowest detection level was 10 CFU/25 g (Table 3 and Fig. 2a–f). Table 3 demonstrates the lowest limit of detection for *Salmonella enterica* serovar Typhimurium. *S*. Typhimurium was not detected in the DBP feed. Fig. 2b and d, which represent the Agilent gel image of the multiplex PCR, demonstrated that the detection level of the PCR for OT and WB was 10 CFU/25 g. Conversely, *Salmonella* identification and serotyping was not achieved in the DBP (Fig. 2f).

**Fig. 3:**
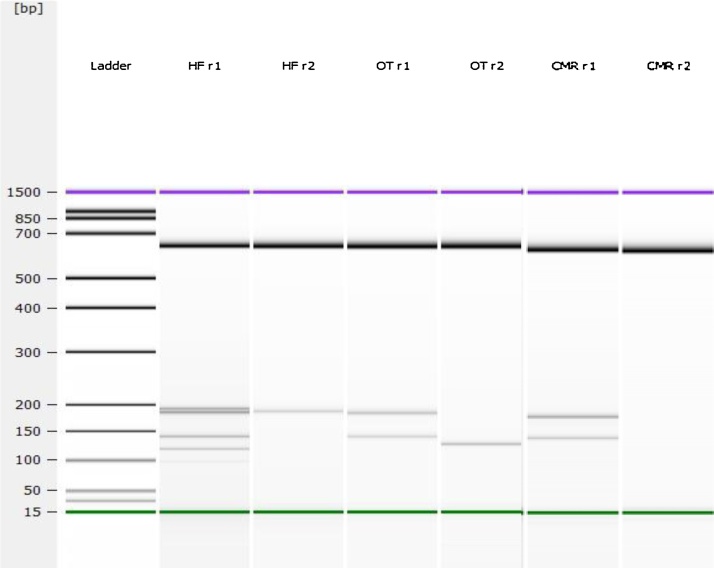
Serotyping reaction 1, and 2 demonstrated in the Agilent 2100 Bioanalyzer gel image for the various *Salmonella enterica* serovars: (Lane 1) Ladder; (Lane 2–3) Typhimurium from Horse Feed with serotypic banding pattern ABCDEI; (Lane 4–5) Agona for Whole Oats with serotypic banding pattern BCJ; (Lane 6–7) Hadar for Calf Milk Replacer with serotypic banding pattern BC.

**Fig. 4:**
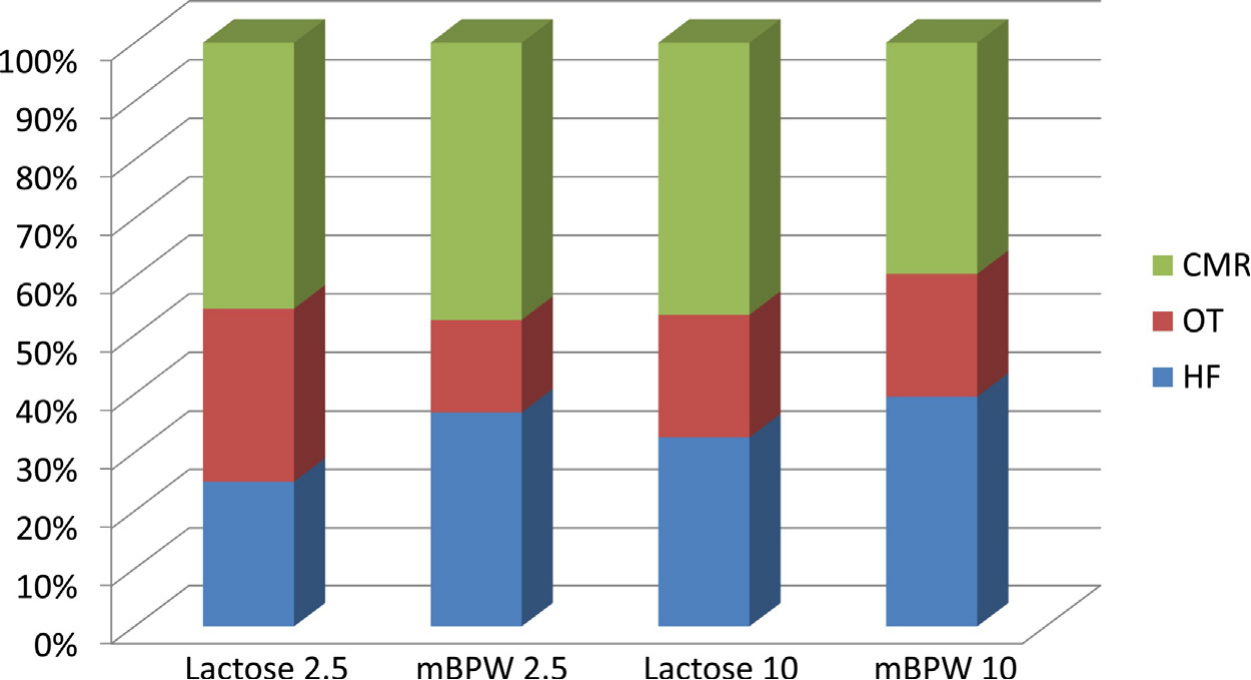
A graph representation of the growth difference of Typhimurium in Horse Feed (HF), Agona in Whole Oats (OT), Hadar in Calf Milk Replacer (CMR) at 2.5 CFU/25 g and 10 CFU/25 g concentration in lactose versus mBPW.

The PCR assay was further evaluated in Horse Feed, Whole Oats, and Calf Milk Replacer spiked with two concentrations of 10 CFU/25 g and 2.5 CFU/25 g of *Salmonella enterica* serovars Typhimurium, Agona, and Hadar respectively (Table 4). A total of 92 samples were analyzed per feed. For the HF feed there was more positive cultures recovered using the mBPW (14/20 and 17/20) as compared to the lactose (11/20 and 14/20) broth at both the 2.5 CFU and 10 CFU per 25 g concentration (Table 4), which represented almost a 10% difference in recovery (Fig. 4). For the CMR feed there slightly was more positive cultures recovered using the mBPW (18/20 and 20/20) as compared to the lactose (20/20 and 17/20) broth at both the 2.5 CFU and 10 CFU per 25 g concentration (Table 4), but it was not a significant difference, equaling to less than 5% difference in recovery. However for the OT feed there were more positive cultures recovered using the lactose (13/20 and 9/20) as compared to mBPW (6/20 and 9/20) as demonstrated in Table 2. At the 2.5 CFU slightly more than 10% difference in recovery using the lactose broth (Fig. 4). There was not much difference observed with mBPW as compared to the lactose broth at 10 CFU per 25 g concentration (Table 4). The 12 remaining samples were the positive controls of each serovar grown in each enrichment broth and un-inoculated enrichment broths for negative controls the results were as expected. The expected PCR amplicons or serotype banding patterns were obtained for serovars Typhimurium (ABCDEI), Agona (BCJ) and Hadar (BC) [10] at the lowest concentrations of 2.5 CFU/25 g (Table 4, Fig. 3). The microbiological limit of detection in food matrices was 100 CFU/25 g. These results are significant in demonstrating the ability of the multiplex assay to detect *Salmonella* from a pre-enrichment broth with an initial inoculum of 2.5 CFU/25 g. These results also show that the assay is effective for screening in multiple animal feeds, and two different broths. In this study the pre-enrichment broth did not have a negative impact on the PCR assay results. However, the correct broth per feed must be used for optimal *Salmonella* enrichment. The assay is effective to screen for *Salmonella* after 24 h, an earlier time point than the current *Salmonella* method which takes 5–10 days to identify *Salmonella* and the serovar.

**Table 4:** A summary of the results using concentrations 2.5 CFU/25 g and 10 CFU/25 g of in Horse Feed, Whole Oats, and Calf Milk Replacer, spiked with serovars, Typhimurium, Agona, Hadar, respectively.

Matrix	Total Samples (n)	ID/Total Positive in mBPW 2.5–10 CFU/25g	ID/Total Positive in Lactose 2.5–10 CFU/25g	ID/Total Positive in mBPW 10–50 CFU/25g	ID/Total Positive in Lactose 10–50 CFU/25g	Internal Salmonella Positive control in PCR (*inv*A)	Serotypic Banding Patterns^a^	Serovars used in the study
Horse Feed	92	14/20	11/20	17/20	14/20	Positive	ABCDEI	Typhimirium
Whole Oats	92	6/20	13/20	9/20	9/20	Positive	BCJ	Agona
Calf Milk replacer	92	18/20	20/20	20/20	17/20	Positive	BC	Hadar

a Serotypic banding patterns as reported by Jean-Gilles Beaubrun et al. [2].

In summary, since animal feed is a recognized source of *S. enterica* for farm livestock [15], it is also a potential *Salmonella* contamination source. *Salmonella* infections in animals can be due to contaminated feed. Outbreaks of human salmonellosis are sometimes linked to contact with infected animals and animal feed. Therefore, it is important to rapidly and efficiently detect *Salmonella* in feed. In this study, the multiplex PCR for molecular serotyping method was tested in the enrichment step and the results show that this method could prove to be a very useful and effective tool for rapid screening of animal feed for the presence of *Salmonella.* This multiplex assay can identify *Salmonella* directly from the pre-enrichment broth after 24 h, instead of waiting for a pure culture to serotype *Salmonella*, which typically may take 5–10 days for the entire process. This approach was verified in a study reported by Benhamed et al. (2017) for the identification of *Salmonella enterica* serovar Cubana in a naturally incurred chick feed [4], [5]. Further testing in naturally contamined feeds would evaluate the limit of this method. The multiplex PCR serotyping assay may be used for rapid screening and serotyping of *Salmonella* contaminating animal feed, thereby decreasing the time it takes to detect *Salmonella* in animal feed and thus helping to prevent human disease.

## Additional information

### Background

*Salmonella enterica* is a leading cause of food-borne illness and is a serious public health concern. Outbreaks of human salmonellosis are often linked to contact with infected animals and feed, as well as contact while preparing and eating contaminated meat. Global outbreaks of human salmonellosis linked to contaminated feed ingredients have been reported [3]. Crump *et. al*., reported that in 1993, the FDA tested for the presence of *S. enterica* in samples from 78 rendering plants that produced animal protein based animal feed and in samples from 46 feed mills that produced vegetable protein based animal feed. *S. enterica* were detected in 56% of the 101 animal protein based samples and 36% of the 50 vegetable protein based samples. Furthermore, in 1994, the FDA tested 89 finished feed samples collected from feed mills and from farms where animal feed is mixed and found that 25% of the samples were contaminated with *S. enterica* [3].

Animal feed and feed ingredients are important sources of zoonotic *Salmonella* infections [12] and might also act as an indirect source of infection in humans consuming foods of animal origin [3], [10]. Therefore, it is imperative to rapidly and efficiently detect *Salmonella* in feed to reduce or prevent consumption of contaminated food/feed. Identification and removal of contaminated feed will provide a prevention step that will reduce and prevent foodborne illnesses associated with the “farm to fork continuum.” This will help ensure that feed used for food animals is introduced into commerce free of *Salmonella*, especially since livestock may be both direct and indirect sources of both zoonotic and human infections.

Hence, the approach in this investigation is to test the capability of a molecular serotyping scheme previously used to serotype *S. enterica* from food matrices spiked with *S. enterica* serovars Newport, Typhimurium, Javiana and Saintpaul [2], [11] to determine its suitability for screening of *Salmonella* in animal feed. The PCR method is a modified version of the multiplex PCR method reported by Kim et al. [6], [7], [8], [13], and it identifies specific serotypes based on PCR amplification of serotype-specific target genes. Amplification of these gene targets in each *Salmonella* serotype produces a serotype-specific banding pattern [11]. This method can serotype 30 of the most clinically relevant *Salmonella* serotypes. The PCR method consists of two five-plex PCR reactions and one two-plex PCR reaction as described by Jean-Gilles Beaubrun et al. [11]. The most common method of serotyping *Salmonella* isolates is based on the serological discrimination of O (surface polysaccharide), H (flagellar) and Vi (capsular) antigenic properties [6], [7], [8], [9]. The conventional method of serotyping employs more than 150O and H antisera for the characterization of over 2600 *Salmonella* serotypes, of which 1478 belong to the species *S. enterica* [14], [16] and take 5 to 10 days. The multiplex PCR serotyping method can generate a result for the 30 serotypes after 24 h of pre-enrichment in a non-selective broth.

The advantage of this approach is that one PCR reaction can screen, identify and serotype *Salmonella enterica* from various contaminated feed matrices. This approach is significant since *S. enterica* serotypes identification remains a highly important public health concern for the microbiological analysis of foods and animal feeds. The capability of this approach over multiple food and feed may increases the global food safety community’s ability to screen for *Salmonella*, which may lead to the prevention of *Salmonella* infections in animals and outbreaks of human salmonellosis.
